# Tubular Duplication of the Esophagus in a Newborn, Treated by Thoracoscopy

**DOI:** 10.1055/s-0042-1742594

**Published:** 2022-03-10

**Authors:** Igor Khvorostov, Alexey Gusev, Abdumanap Alkhasov, Sergey Yatsyk, Elena D'yakonova

**Affiliations:** 1 Department of Pediatric Surgery, Volgograd State Medical University, Volgograd, Russian Federation; 2 Department of Pediatric Surgery, Federal State Autonomous Institution “National Medical Research Center for children health” MH RF, Moscow, Russian Federation; 3 Department of Pediatric Surgery, RUDN University, Moscow, Russian Federation

**Keywords:** esophageal duplication, newborn, thoracoscopy

## Abstract

We present a case of tubular esophageal duplication in a 3-day-old female newborn (38 weeks, 2,500 g) without concomitant abnormal development. Esophageal duplication was diagnosed based on the clinical picture, direct laryngoscopy, esophagography and computed tomography. The duplicated esophagus was resected by thoracoscopy leaving the orthotopic esophagus in place. Isolation from the pharynx was performed via a separate cervical incision. After a follow-up period of 20 months, the child returned to normal growth and development.

## Introduction

Congenital tubular esophageal duplication is a rare congenital anomaly in which the second esophagus with mucous, submucous, and muscular membranes, corresponding to the esophagus, is adjacent to the true esophagus without a common wall. This condition causes dysphagia, nausea, vomiting, retrosternal pain or respiratory problems (stridor and recurrent pneumonia). Presentation typically occurs during the newborn period. Tubular esophageal duplication represents approximately 10% of all foregut duplications. 1 2 The incidence of this malformation is estimated to be 1:8,200, with male sex predominance 2:1. 3 It is subgrouped into three types as follows: (1) cystic (the most common), (2) tubular, and (3) diverticular. Very few cases have been reported and described in newborns. 4 5 6 7 8

## Case Presentation

**Fig. 1:**
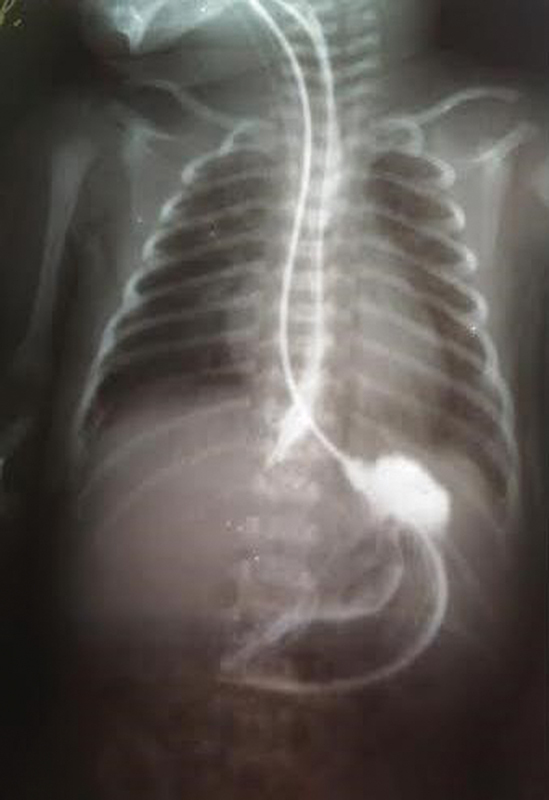
Esophagram via gastrografin swallow showing tubular esophageal duplication extending from the neck to the diaphragm.

**Fig. 2:**
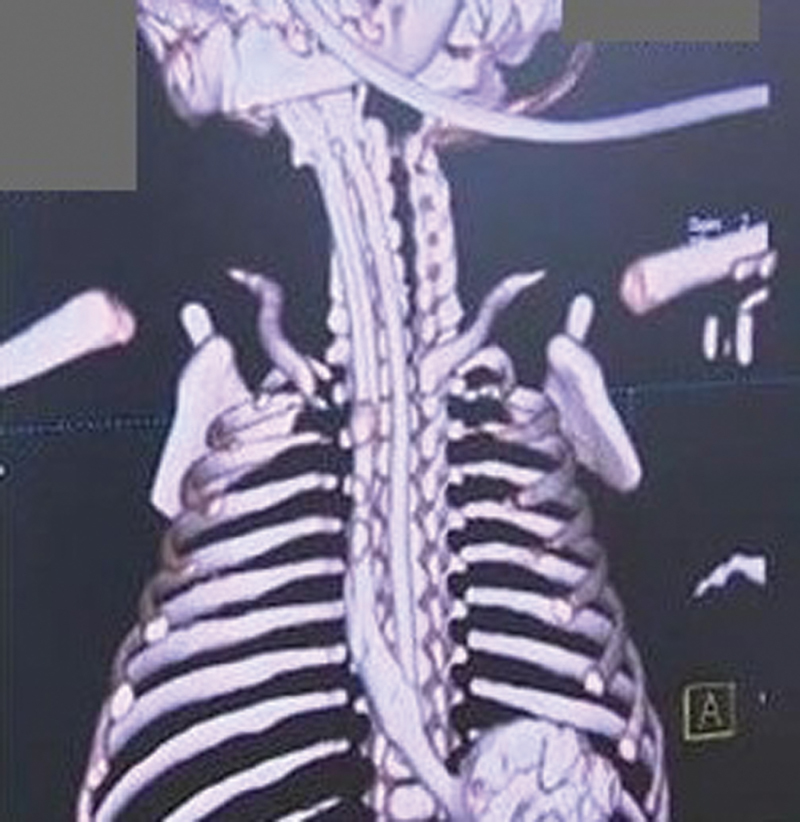
Computed tomography (CT) showing a second lumen along right the orthotopic esophagus.

**Fig. 3:**
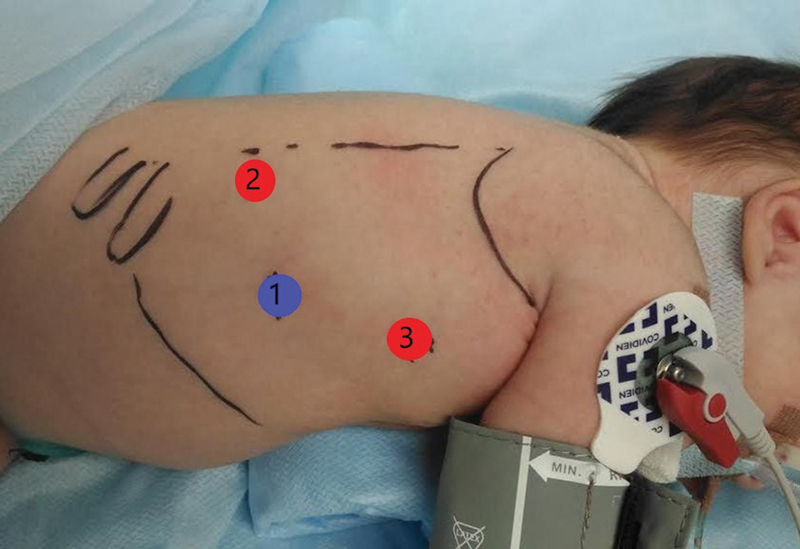
Patient positioning and placement of trocars: 1, camera; 2 and 3, working ports.

**Fig. 4:**
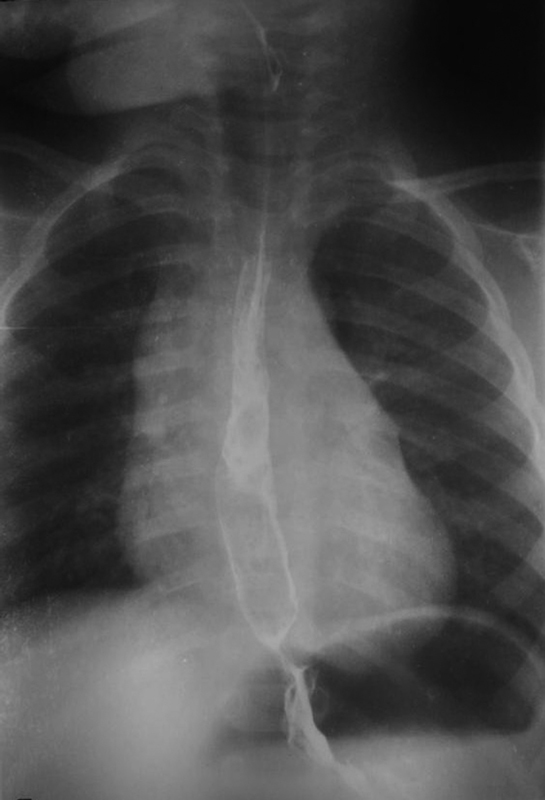
Follow-up contrast esophagram 1 year after surgery showing a normal position of the orthotopic esophagus.

We report the case of a 3-day-old female (38 weeks and 2,500 g) presented to our surgical clinic from the maternity hospital with a history of respiratory distress, salivation, and dysphagia. The newborn was delivered via caesarean section to a primigravida mother. There was no visible additional malformation. On a physical examination in maternity hospital, she had tachycardia and tachypnea, maintaining oxygen saturation of 88% at room air. The patient aspirated and was intubated and ventilated, a nasogastric tube was placed. The child was consulted by a surgeon and transferred to a surgical clinic for further examination. During laryngoscopy, two esophageal lumen were discovered. Two nasogastric tubes were placed. First into the blind-ending esophagus and second into the normal esophagus under fluoroscopic guidance. The nasogastric tube was used for enteral feeding while the tube in the long and blind-ending duplicated esophageal pouch was attached to low pressure suction pump. A contrast esophagram confirmed a tubular esophageal duplication extending from the cervical region to the diaphragm without any gastric communication. The duplicated segment was found on the left side in the neck and on the right posterior side of the normal esophagus in the thorax ( Fig. 1 ). Computed tomography (CT) confirmed a tiny duplicated lumen to the right of and along the orthotopic esophagus ( Fig. 2 ). А Nelaton's catheter was inserted into the double esophagus before the operation for better visualization of the tubular duplication esophagus during thoracoscopy. The infant underwent a right thoracoscopy. The procedure was performed with three ports. The initial 5-mm trocar was inserted just below the right scapula tip and a 5-mm camera was inserted as well. А 3-mm port was placed in the fifth intercostal space posterior to the tip of the scapula. Another 3-mm trocar was inserted at the right midaxillary line along the scapula margin ( Fig. 3 ). The esophageal duplication tubular sac was removed from the surrounding tissue all the way from the main esophagus using a coagulation hook and monopolar coagulation. There was no common wall between the tubular and the main esophagus. The dissection of the doubled esophagus was continued cranially to the level of the superior chest aperture. Then, a left cervical neck incision was made and the tubular esophagus was removed. The child was left intubated for 5 days. Feeds via the nasogastric tube were commenced up until the sixth postoperative day. Recovery of the child was uneventful and the patient was discharged on the postoperative day 16. Histopathological examination showed a gastric-type mucosa with a well-developed sub mucosa, muscularis propria, and serosa. A contrast esophagram via gastrografin swallowed 2 weeks after surgery showed a normal esophagus ( Fig. 4 ). After a follow-up period of 20 months, the child returned to normal growth and development.

## Discussion

Pediatric tubular duplication of the esophagus is a rare congenital anomaly. The incidence of congenital esophageal duplication is estimated to be 1:8,200, with male sex predominance (male:female = 2:1). 9 Cystic duplications are far more common, with tubular duplications accounting for less than 10% of cases. 10 Tubular duplications without communication to the normal esophagus are more common than cystic duplications. Classically, these patients suffer from gastrointestinal and respiratory symptoms like nausea, vomiting, dysphagia, respiratory distress, or aspiration pneumonia. Diagnosis can be made by esophageal contrast study, with several duplications discovered incidentally on X-ray of the chest. A CT can be useful in making a diagnosis by showing a second tubular structure adjacent to the esophagus. In this case, the diagnosis was made via upper endoscopy. It allowed delineating the detailed anatomy, the extent of duplication, presence and length of a common wall, and a possible communication to the normal esophagus or trachea. It is well documented that a surgical excision is a viable option for the duplication of the esophagus. 4 11 In our patient, the thoracoscopic approach was successful. This report shows that although extremely rare, a tubular esophageal duplication should be considered as a differential diagnosis in newborns with feeding intolerance. After surgical excision of the additional esophagus, prognosis is favorable in most cases. 9 12

## References

[1] Alenazi F Alenazi E Asaad Z Alqoaer K Tubular duplication of the esophagus case reports in internal medicine Singapore Med J 2017 4 01 46 49

[2] Herbella F A Tedesco P Muthusamy R Patti M G Thoracoscopic resection of esophageal duplication cysts Dis Esophagus 2006 19 02 132 134 16643183 10.1111/j.1442-2050.2006.00552.x

[3] Kapoor K Jajoo M Dublish S Mohta A A rare cause of wheezing in an infant: Esophageal duplication cyst Indian J Crit Care Med 2014 18 07 464 466 25097361 10.4103/0972-5229.136077 PMC4118514

[4] Garge S Samujh R Isolated complete tubular esophageal duplication in a neonate Dis Esophagus 2013 26 03 342 22486784 10.1111/j.1442-2050.2012.01338.x

[5] Huang Y Wang D Liu X Wang W Zhang Z Communicating esophageal tubular duplication in a newborn infant J Pediatr Surg 2011 46 08 1655 1657 21843739 10.1016/j.jpedsurg.2011.04.060

[6] Kim J H Kwon C I Rho J Y Communicating tubular esophageal duplication combined with bronchoesophageal fistula Clin Endosc 2016 49 01 81 85 26855929 10.5946/ce.2016.49.1.81 PMC4743716

[7] Lima M Molinaro F Ruggeri G Gargano T Randi B Role of mini-invasive surgery in the treatment of enteric duplications in paediatric age: a survey of 15 years Pediatr Med Chir 2012 34 05 217 222 23342745 10.4081/pmc.2012.57

[8] Morán Penco J M Vázquez J Forsheden Ahs E Sanjuán Rodríguez S Pairola A García-Martínez V Complete and inverted esophagastric duplicity [in Spanish] Cir Pediatr 2017 30 03 169 171 29043696

[9] Cuch B Nachulewicz P Wieczorek A P Wozniak M Pac-Kozuchowska E Esophageal duplication cyst treated thoracoscopically during the neonatal period: clinical case report Medicine (Baltimore) 2015 94 49 e2270 26656375 10.1097/MD.0000000000002270 PMC5008520

[10] Trappey A F III Hirose S Esophageal duplication and congenital esophageal stenosis Semin Pediatr Surg 2017 26 02 78 86 28550875 10.1053/j.sempedsurg.2017.02.003

[11] Barabino A Nardi F Arrigo S Tubular esophageal duplication: further evidence of a possible endoscopic treatment J Pediatr Gastroenterol Nutr 2014 58 06 e53 22874428 10.1097/MPG.0b013e31826c21f6

[12] Scarpa A A Ram A D Soccorso G Singh M Parikh D Surgical experience and learning points in the management of foregut duplication cysts Eur J Pediatr Surg 2018 28 06 515 521 29069668 10.1055/s-0037-1607293

